# Recent Progress in Biosensors for Detection of Tumor Biomarkers

**DOI:** 10.3390/molecules27217327

**Published:** 2022-10-28

**Authors:** Mantong Li, Feng Jiang, Liangyi Xue, Cheng Peng, Zhengzheng Shi, Zheng Zhang, Jia Li, Yupeng Pan, Xinya Wang, Chunqiong Feng, Dongfang Qiao, Zhenzhong Chen, Qizhi Luo, Xuncai Chen

**Affiliations:** 1 Department of Forensic Toxicology, School of Forensic Medicine, Southern Medical University, Guangzhou 510515, China; 2 Guangzhou Key Laboratory of Forensic Multi-Omics for Precision Identification, School of Forensic Medicine, Southern Medical University, Guangzhou 510515, China; 3 Guangzhou Institute of Food Inspection, Guangzhou 510080, China

**Keywords:** cancer, tumor biomarker, biosensor, detection, nanomaterial

## Abstract

Cancer is a leading cause of death worldwide, with an increasing mortality rate over the past years. The early detection of cancer contributes to early diagnosis and subsequent treatment. How to detect early cancer has become one of the hot research directions of cancer. Tumor biomarkers, biochemical parameters for reflecting cancer occurrence and progression have caused much attention in cancer early detection. Due to high sensitivity, convenience and low cost, biosensors have been largely developed to detect tumor biomarkers. This review describes the application of various biosensors in detecting tumor markers. Firstly, several typical tumor makers, such as neuron-specific enolase (NSE), carcinoembryonic antigen (CEA), prostate-specific antigen (PSA), squamous cell carcinoma antigen (SCCA), carbohydrate, antigen19-9 (CA19-9) and tumor suppressor p53 (TP53), which may be helpful for early cancer detection in the clinic, are briefly described. Then, various biosensors, mainly focusing on electrochemical biosensors, optical biosensors, photoelectrochemical biosensors, piezoelectric biosensors and aptamer sensors, are discussed. Specifically, the operation principles of biosensors, nanomaterials used in biosensors and the application of biosensors in tumor marker detection have been comprehensively reviewed and provided. Lastly, the challenges and prospects for developing effective biosensors for early cancer diagnosis are discussed.

## 1. Introduction

As a leading life-threatening disease, cancer has a prominent effect on a large scale and causes cancer-related mortality to increase rapidly. Due to the poor diagnosis and prognosis at the early stage, the mortality of cancer patients remains at a high level. At present, many methods are being used to detect early tumors, such as computed tomography (CT), chest radiograph (CRG), magnetic resonance imaging (MRI) and positron emission tomography (PET), as well as a biopsy [1]. It should be noted that a biopsy is still the diagnostic criteria of tumors. In recent years, methods based on biochemistry, immunology and molecular biology have been developed for the determination of tumor markers in serum [2]. Immunoassay techniques such as radioimmunoassay and enzyme-linked immunosorbent assay (ELISA) have become the main methods in the clinical quantitative detection of tumor markers, but these methods still have disadvantages, such as being time-consuming, the high cost and the requirement of qualified personnel and sophisticated instrumentation [2].

It is worth mentioning that serum tumor markers are produced by the reaction of tumor tissue or host to tumor, which are proteins related to malignant tumors. They can reflect and evaluate the occurrence and development of tumors and then predict tumor progression. Thus, they can be used in the clinical diagnosis of tumor patients and can also be used to evaluate the clinical efficacy of tumor patients by monitoring the changes of tumor markers [3]. Therefore, the subtle detection of tumor markers has important clinical application value.

**Figure 1:**
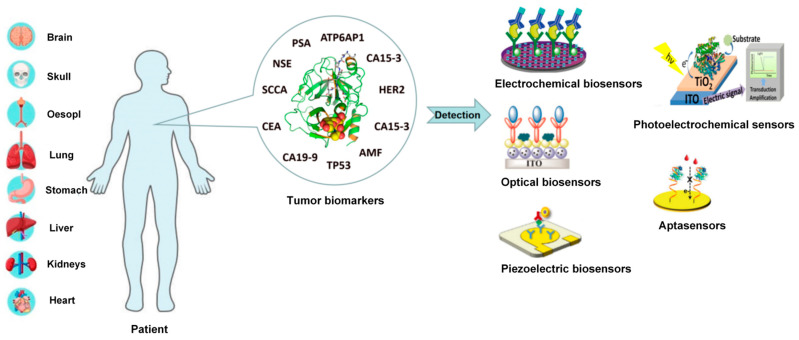
Schematic illustration of the application of electrochemical, optical, photoelectrochemical, piezoelectric sensors/biosensors and aptasensors for the detection of tumor biomarkers in a cancer patient.

Due to the high sensitivity, convenience and low cost, biosensors have been largely developed to detect tumor biomarkers in the past decade. The biosensor is a kind of signal technology based on the specific combination of biomolecules and target analytes to read invisible biological reactions [4]. There are many kinds of biosensors, which are mainly classified as electrochemical, optical, photoelectrochemical biosensors, piezoelectric biosensors and aptasensors. The biosensor is composed of a recognition element and a signal converter, which can recognize the detection object group in a specific sample as the specificity, convert it into a readable signal and output it. The recognition element, also known as turbine acceptor, is an important part of biosensors. The signal converter includes potential measuring electrodes, piezoelectric crystals and current sensors. Electrochemical biosensors detect the generation or consumption of electroactive substances according to the principle of potential, current or resistance conversion and indirectly reflect the concentration of the measured object. Optical biosensors use the interaction between analyte and converter in optical properties such as absorption, luminescence, fluorescence, reflectance and surface plasmon resonance (SPR) for quantitative or qualitative detection. Photoelectrochemical sensors are a combination of the advantages of electrochemical and optical biosensors. The piezoelectric sensor uses the resonance generated by the external application of alternating the current and used electrical components and other devices to convert the measured pressure into electricity for related detection actions. In the previous descriptions of biosensors, most of them used antibodies as recognition elements, while the use of other novel biomolecules instead of antibodies is a new strategy for the current biosensor design. Aptamer sensors use single-stranded oligonucleotides that can bind to proteins or small molecules as recognition elements. These five biosensors have been largely studied and applied for the detection of tumor biomarkers, which have presented advantages of rapid, low-cost, high selectivity and sensitivity and easy operation in complex samples. With the rapid development of nanomaterials, many ultrasensitive biosensors have been developed for the detection of tumor biomarkers. Thus, it is necessary to summarize the recent research developments on biosensors used for tumor biomarkers detection. In this review, we firstly introduce various typical tumor biomarkers and comprehensively summarize five biosensors, including electrochemical biosensors, optical biosensors, photoelectrochemical sensors, piezoelectric biosensors and aptasensors, as shown in Figure 1. Specifically, the working principle, used nanomaterials and application of these five biosensors are systematically introduced. Finally, to develop effective biosensors for the early diagnosis of cancer, the recent challenges and further opportunities are discussed.

## 2. Tumor Biomarkers

Tumor biomarkers and biochemical parameters for reflecting cancer occurrence and progression have caused much attention in early cancer detection. There are too many kinds of tumor biomarkers to generalize all of them. Hence, the most effective approach is to select several representative biomarkers and describe them. As shown in Table 1, different cancers present various associated tumor markers. In this review, six typical tumor biomarkers, including NSE, CEA, PSA, SCCA, CA19-9 and TP53, will be introduced circumstantially.

NSE is an acid protease specific to neurons and neuroendocrine cells. It is a specific marker of neuroendocrine tumors, such as neuroblastoma, medullary thyroid carcinoma and small cell lung cancer (70% increased).

CEA is a common tumor marker in gastrointestinal tumors [5]. Most (70–90%) patients with colon adenocarcinoma are highly positive for CEA, and 53% of patients with gastric cancer are positive for CEA. The concentration of CEA is related to tumor size and metastasis, especially when there is liver metastasis [6].

PSA exists in many kinds of tissues and body fluids in the human body. The concentration of PSA expression in prostate tissue and semen is the highest, which is one of the most abundant proteases in semen. In normal prostate tissue, there is a barrier between the epithelial cells of the duct and the blood. When prostate cancer occurs, the lesion destroys the border between the blood and the epithelium. Thus, the PSA secreted by the cancerous prostate tissue increases significantly, resulting in a large PSA entering the blood circulation [7].

SCCA plays an important role in the diagnosis, condition analysis, curative effect judgment and prognosis of a variety of squamous cell carcinoma, including cervical cancer, lung cancer, liver cancer, a urogenital system tumor and so on. Many studies have shown that serum SCCA plays a crucial role in the diagnosing, classifying and staging of cervical cancer [8].

CA19-9 widely exists in normal human digestive system tumor tissues, such as the stomach, colon, pancreas and gallbladder. CA19-9 also exists in other system tumor tissues, such as lung cancer, thyroid cancer and breast cancer [9].

TP53 is a gene that regulates the cell cycle by encoding a protein, which has the effect of inhibiting a tumor. Therefore, tumor cells lacking p53 can tolerate genomic instability and enhanced carcinogenic signal transduction, which is a sign of malignant transformation [10]. According to the relevant research, p53 has great potential as a biomarker for breast cancer, prostate cancer and other cancers.

It is worth mentioning that, in the diagnosis of whether a person has cancer or has recovered from cancer, the content of the associated tumor markers should be quantitatively analyzed first and then evaluated by comparing them with their corresponding thresholds. The thresholds of various tumor markers have been listed in Table 2. In other words, the accurate and efficient detection of tumor markers is crucial for cancer diagnosis and prognosis.

## 3. Classification of Biosensors

### 3.1. Electrochemical Biosensors

Electrochemical biosensors have been applied in many fields, such as biomedical analysis, food manufacturing and environmental monitoring. The cheap and portable electrodes endow the electrochemical biosensors with the advantage of measuring the target analyte quickly in a miniaturized portable system. In addition, the concentration of the target analyte could be determined even in complex samples, which is beneficial to the medical diagnosis, environmental monitoring and existing condition monitoring [11]. Recently, electrochemical biosensors have been widely used to detect and quantify biomarkers. The advantages, such as quick response, accurate quantification, universality, multiplexing and miniaturization, make electrochemical biosensors have a promising future in detecting tumor markers [12].

#### 3.1.1. Principles of Electrochemical Biosensors

**Figure 2:**
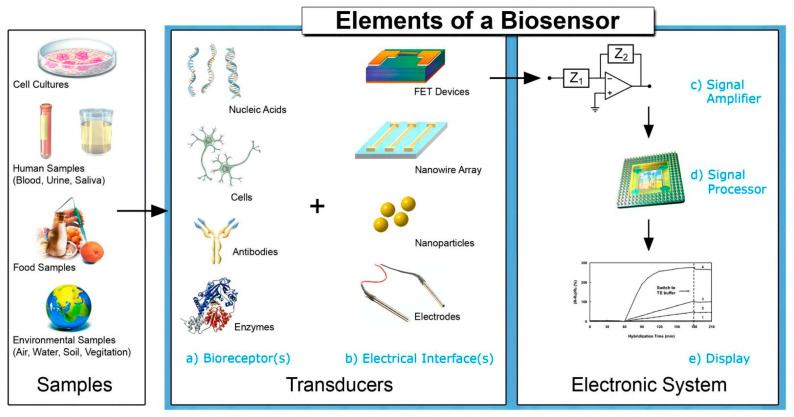
Elements and selected components of a typical biosensor. Reprinted with permission from [19].

Electrochemical biosensors work by converting biochemical reactions such as an enzyme–substrate reaction and antigen–antibody interaction into electrical signals (such as current, voltage, impedance, etc.). In other words, the electrochemical biosensor detects the generation or consumption of electroactive substances based on the principle of potential, current or resistance conversion, thus indirectly reflecting the concentration of the measured object. Figure 2 shows the working principle of an electrochemical biosensor. In the field of electrochemical biosensing, various responses can be produced based on different electrochemical techniques. The common electrical analysis techniques include voltammetry, amperometry, potentiometry and electrochemical impedance spectroscopy (EIS) [13]. Voltammetry and the ampere method can form over the potential by controlling the fixed or variable potential of the whole electrochemical cell. After the overpotential is formed, electron transfer becomes feasible in thermodynamics, and the oxidation or reduction reaction will take place [11]. The potential biosensor is a kind of equipment containing a biosensing element, which is connected to the electrochemical sensor, and its analysis signal is the potential [14]. EIS measures the resistance and capacitance characteristics of the interface after interfering with the system with a small amplitude (about 2–10 mV) sinusoidal AC excitation signal. Change the frequency in a wide range to obtain the impedance spectrum [15].

It is worth mentioning that the electrode is the key component of an electrochemical biosensor, which acts as a solid carrier for immobilizing biomolecules (enzymes, nucleic acids or antibodies) and electron movement. Therefore, the performance of biosensors can be significantly improved by using appropriate electrode functional materials. According to the recently published research, the most commonly used functional materials are metal nanoparticles, carbon-based nanomaterials and their hybrid composites. In the field of high-sensitivity electrochemical biosensors, metal nanomaterials have always been very attractive; among which, gold nanoparticles (AuNPs) have high conductivity, high affinity and compatibility with biomolecules, so AuNPs are the most widely used metal nanoparticles to construct electrochemical biosensors [16]. In addition, among the carbon-based nanomaterials, carbon nanotubes have high mechanical strength, thermal conductivity and electrical conductivity, so they are often used to construct biosensors [17]. Finally, the hybrid composite of the above materials can give full play to its advantages and optimize the performance of the sensor. Common electrochemical biosensors based on these functional materials will be described in detail below [18].

#### 3.1.2. Electrochemical Biosensors Based on Nanomaterials

Metal nanoparticles, such as Au and Ag nanoparticles, have been widely developed and coated on the electrode surface used in electrochemical biosensors for tumor biomarker detection. Many methods that have been used to modify the electrode surface of electrochemical biosensors with metal nanoparticles. For example, electrodeposition, a process that uses the electric current to reduce dissolved metal cations to form a coherent metal coating on an electrode, was employed to coat gold nanoparticles on the surface of the electrode. Furthermore, the AuNP-modified electrode was further modified with antibodies to prepare an electrochemical immunosensor. In addition, the AuNP can be deposited on the electrode by dip coating, drop casting or multilayer deposition. For example, the preparation of a sensitive immunosensor to prostate-specific antigen (PSA) labeled by prostate cancer is achieved by fixing anti-prostate-specific antigen on AuNP.

Graphene, carbon nanotubes (CNTs) and diamond-like carbon nanomaterials have been widely used in the field of electrochemical sensing due to their excellent properties, such as a large specific surface area, high electrical conductivity and electron mobility at room temperature. For example, graphene, which has high charge transport and electron mobility properties due to its unique electronic band structure, is the most common carbon nanomaterial that could improve the conductivity and stability of the immunosensor. In particular, a vast surface area (2630 m^2^/g) of graphene enables it to interact directly with a wide range of biomolecules. These unique properties of graphene are employed and integrated into biosensors for tumor biomarker detection.

In addition to graphene, carbon nanotubes (CNTs) are also common platforms for signal amplification. CNTs have high mechanical strength, electrical conductivity and thermal conductivity, resulting in their wide use in nanotechnology. According to the research results, the carbon nanotube-modified field effect transistor has become a promising device for constructing biosensor platforms. For example, Justino and his colleagues made a C-reactive protein (CRP) sensor using a CNT-modified electrode. The sensor exhibited high sensitivity and an extensive detection range from CRP molecules after being exposed to the CRP solution [20].

Recently, mixed composites of metal nanoparticles and carbon nanomaterials are increasingly causing much attention. For example, a functionalized gold-graphene oxide nanocomposite electrode for an electrochemical immunosensor was proposed by Sharma et al. In their study, it was concluded that the functional GO with good electrical properties was conducive to the better detection of an electrochemical signal for diuron. In addition, Zahra et al. proposed an electrochemical biosensor by using modified graphene oxide–gold nanostructures to detect the total and free PSA antigens. The specificity of antibody recognition and high binding affinity significantly improved the selectivity and sensitivity of the sensor that is expected to be used as a diagnostic tool for PSA tumor marker detection and clinical analysis [21].

### 3.2. Optical Biosensors

Similar to electrochemical biosensors, optical biosensors have the advantages of high specificity, high sensitivity and low cost and are widely used in many fields, such as the environment, medicine and biotechnology. There is a great practical significance and development prospect for optical biosensors.

#### 3.2.1. Principles of Optical Biosensors

**Figure 3:**
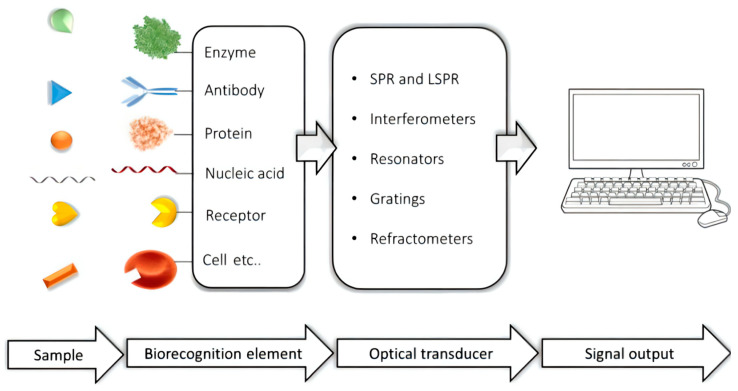
The working principle of optical biosensors. Reprinted with permission from [22].

Optical biosensors are the most common kind of biosensors. They use the change of the optical signal (such as fluorescence, color and refractive index change) caused by the reaction of the test substance and detection reagents as the detection basis. In another words, it is based on the interaction between optical fields and biometric elements for the optical detection and conversion of optical signals into electrical signals to detect the target analyte. Generally, an optical biosensor is composed of three functional modules: sensing layer, optical signal conversion and amplification processing (Figure 3). Optical biosensors are often used in food detection, environmental monitoring, medical fields and biotechnology fields; among which, the most widely used is in the medical field. They can be used for genetic analysis, genetic testing, protein testing and drug screening. Cancer has always been an important problem in the field of medicine. Thus, this review will introduce the detection of common tumor markers such as CEA, NSE, PSA and SCCA by optical biosensors.

#### 3.2.2. Surface-Enhanced Raman Scattering (SERS)

Surface-enhanced Raman scattering (SERS), a spectroscopic technique that combines modern laser spectroscopy with the exciting optical properties of metallic nanostructures, has been considered as the most promising optical biosensor for biomarker detection in recent years. The basic principle of Raman spectroscopy is a type of vibrational spectroscopy that relies on the inelastic scattering of laser photons, which reveals information about the molecular structure of samples. Due to the low sensitivity, the application of Raman spectroscopy in the biomedical field was hindered [23]. In recent years, surface-enhanced Raman scattering has been developed and applied as optical biosensors. Surface-enhanced Raman scattering exhibits a strong Raman signal due to the presence of plasma nanostructures such as a metal colloid; this is caused by both electromagnetic enhancement and chemical mechanisms [24]. Typically, a Raman spectrometer consists of a laser (light source), an out-of-sample light path, a monochromator, an amplifier, a detector, and a controller. In particular, nanoparticles were used to promote surface-enhanced Raman scattering (SERS), and the Raman intensity showed strong enhancement (typical increase range was 10^6^–10^12^ times), which was very suitable for biomedical application [25].

### 3.3. Photoelectrochemical Biosensors

A photoelectrochemical sensor is a new type of sensor that combines an optical sensor and electrochemical sensor skillfully. It is one of the hotspots of the current research because of its simplicity, fast speed, low detection limit and high sensitivity.

#### Principle of Photoelectrochemical Biosensors

Its working principle is that, in photoelectrochemical detection, light is irradiated on the surface of the photosensitive material to generate electrons and holes, causing changes in the electrical signals. The electrical signal is converted into a detection signal that can be read out, and the quantitative relationship between the analyte and the photocurrent or photovoltage is carried out by analyzing the signal. Using the optical signal as the excitation source can be completely separated from the current or voltage as the detection signal, which greatly reduces the interference of the background signal. The mechanism of photoelectric generation is a redox reaction in the solution. The photocurrent is divided into two forms; one is the anode photocurrent, and the other is the cathode photocurrent. Photoelectric materials can be divided into inorganic semiconductor materials, such as Si, TiO_2_, CdS, etc., and organic photoelectric molecules, such as organic small molecule photoelectric materials, composite materials, etc. Among them, TiO_2_-based composite materials are currently studied more because of their higher photoelectric conversion efficiency. PEC sensors are usually composed of three parts: light source, optical path and optoelectronic components. The excitation light sources are divided into physical light sources (ultraviolet and visible light), chemiluminescence and electrochemiluminescence. Nevertheless, short-wavelength ultraviolet light will damage biological materials, so attention should be paid to the selection.

### 3.4. Piezoelectric Biosensors

Piezoelectric sensors are widely used in biosensor detection and analysis because of their diversity of piezoelectric materials and simple affinity interaction.

#### 3.4.1. Principles of Piezoelectric Biosensors

**Figure 4:**
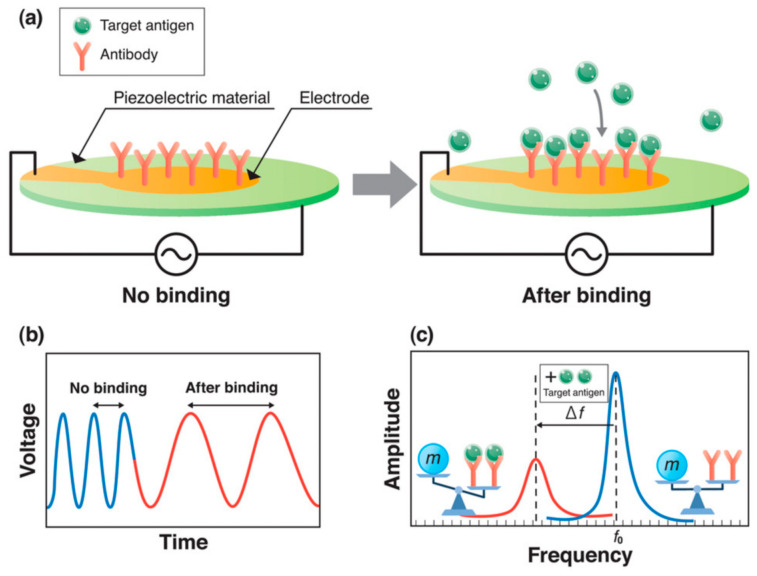
(**a**) The working principle of the piezoelectric biosensor. (**b**) Influence of voltage on time. (**c**) The influence of amplitude on frequency. Reprinted with permission from [27].

The piezoelectric biosensor is a kind of micromechanical sensor characterized by using piezoelectric crystal material as an electrode. The working principle of the piezoelectric sensor is shown in Figure 4. Under the excitation of the AC voltage, mass restraint is formed on the surface of the piezoelectric crystal, and then, oscillation changes are produced [26]. Typical piezoelectric materials are anisotropic crystals; that is, crystals without symmetrical centers, such as aluminum phosphate, aluminum nitride, zinc oxide and so on.

The piezoelectric biosensor as an effective analytical tool has been widely used to detect biomarkers in genetic diseases, owing to its good characteristics of rapidity, high sensitivity and low interference [26]. In addition, piezoelectric sensors show good properties in the field of fungal pathogen detection. For example, Fumio Narita et al. found that the sensor had certain potential in detecting the novel coronavirus [27]. In this paper, piezoelectric sensors used in tumor marker detection will be comprehensively discussed.

#### 3.4.2. Quartz Crystal Microbalance (QCM)

Quartz crystal microbalance is one of the piezoelectric sensors. The most basic principle is to use the piezoelectric effect of quartz crystals. A quartz crystal vibrates when an alternating voltage is applied to its two electrodes, but this vibration is usually minimal. However, when the ac voltage is applied at a certain frequency, the vibration effect increases significantly, which is called piezoelectric resonance. When the oscillation frequency of the circuit is equal to the resonance frequency of the quartz crystal oscillation plate, the frequency can be converted into electrical signals to visualize the vibration frequency.

The QCM is mainly composed of a quartz crystal sensor, signal detection and data processing. In clinical medicine, it is often used in combination with the immunoassay known as the quartz crystal microbalance immunoassay. Typically, to amplify the signal, gold nanoparticles are attached to the surface of a quartz crystal, making the results more sensitive. The quartz crystal microbalance (QCM) immunosensor has the advantages of high sensitivity, real-time output, no labeling and low cost [28], which can detect proteins in human plasma, such as tumor markers. It has become a hot spot in clinical research and is expected to be applied in clinical detection in the future.

### 3.5. Aptasensors

Aptasensors are based on the folding of induced binding oligonucleotide aptamers and can be used to detect proteins, small molecules and inorganic ions. Aptamers are specific sequences of nucleic acids with unique binding sites for their targets [29].

#### Principles of Aptasensors

As one of the current research hotspots, the aptamer sensor is mainly composed of three parts: recognition elements, transducing elements and signal transducing elements. At present, the aptasensors are mainly classified into three categories: electrical, optical and electrochemical. Among them, the most commonly used method is the electrochemical method. The aptamer is immobilized on the electrode surface as a biorecognition element in electrochemical aptasensors, and the potential response generated by the oxidation and reduction reactions of the electrode surface is evaluated by specific binding to the target, analyte or current or by evaluating the potential response. According to the type of response signal, it can be divided into the amperometric method, cyclic voltammetry, electrical impedance method, etc. [30]. Among them, the electrochemical aptasensors proposed by Han et al. mainly have four structures, namely the target-induced structure switching mode, sandwich or sandwich-like mode, target-induced dissociation or displacement and competitive replacement mode [29]. Aptamer sensors are not limited by cell lines or animals, can be used for toxic or nonimmunogenic targets, can be repeatedly synthesized in large quantities, have good purity and have the advantage of high stability. Given specific aptasensors for a specific target, they can be modified with functional groups such as fluorophores, nanoparticles or enzymes to enhance their selectivity and sensitivity while maintaining their affinity. The main applications are disease monitoring, targeted therapy, drug analysis and other directions.

## 4. Application of Biosensor in Detection of Tumor Biomarkers

### 4.1. NSE Detection

Human lung cancer is one of the diseases with high cancer mortality globally, which can be divided into small cell carcinoma and non-small cell carcinoma. According to the relevant literature, neuron-specific enolase (NSE) can be used as a tumor marker with high expression in non-small cell lung cancer. However, the reported immunobioluminescence, mass spectrometry and other methods have high requirements on the experimental conditions, complexity and time-consuming pretreatment, so they cannot be widely used.

#### 4.1.1. NSE Detection Based on Electrochemical Biosensors

**Figure 5:**
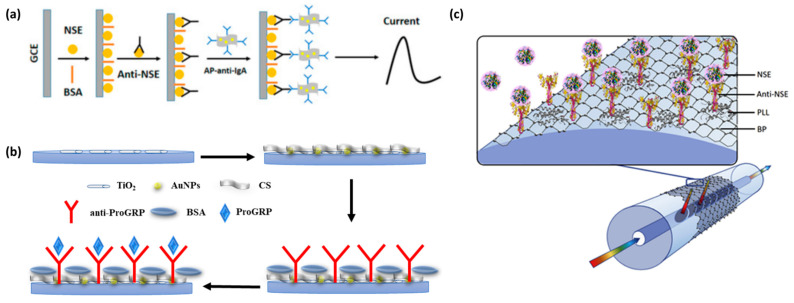
(**a**) Schematic diagram of an electrochemical immunosensor for NSE detection. Reprinted with permission from [37]. (**b**) Schematic diagram of an electrochemical immunosensor structure. Reprinted with permission from [38]. (**c**) Schematic of a biofunctionalized black phosphorus-based fiber optic biosensor. Reprinted with permission from [32].

Kevin Z et al. used gold nanoparticles/reduced graphene oxide composite (AuNP-RGO) as a functional material to modify the electrode and created an unlabeled signal-enhanced electrochemical immunosensor for the detection of neuron-specific enolase (NSE), as shown in Figure 5a. At the same time, they observed that the DPV signal of AP-anti-igg/AUNP-RGO as the detection probe was significantly increased to 15.41 μA (Figure 5b), indicating that AP-anti-igg/AUNP-RGO could significantly improve the intensity and sensitivity of the immunosensor to detect NSE. Under the optimal conditions, the linear relationship between NSE and DPV was in the range of 0.1–0.2 μg/mL, the correlation coefficient was 0.989 and the detection limit was 0.05 ng/mL. The results showed that this neuron-specific enolase immunosensor had a large capacity and high sensitivity and could be applied in practice.

#### 4.1.2. NSE Detection Based on Optical Biosensors

NSE is an acid protease specific to neurons and neuroendocrine cells. It is a specific marker for neuroendocrine tumors, such as neuroblastoma and small cell lung cancer, and can be used for differential diagnosis, disease monitoring, efficacy evaluation and recurrence prediction. For example, Li et al. synthesized a three-dimensional (3D) hyperbranched TiO_2_ nanorod array and used it for the first time to prepare a dopamine (DA)-sensitized photoelectrochemical biosensor [31]. In their work, DA was used as a sensitizer and combined with TiO_2_ to achieve the effect of signal amplification. This biosensor for the determination of the NSE exhibited an excellent linear relationship range from 0.1 ng/mL to 1000 ng/mL, with a detection limit of 0.05 ng/mL. Zhou et al. developed the first black phosphorus (BP) fiberoptic biosensor for the ultrasensitive diagnosis of human NSE cancer biomarkers (Figure 5c) [32]. The method was extremely sensitive, with a LOD of 1.0 pg/mL, 100 times more sensitive than other substance-based biosensors.

#### 4.1.3. NSE Detection Based on Photoelectrochemical Biosensors

In addition to being highly expressed in SCLC, NSE also has immune activity in neuroendocrine tumors and has been used as a very useful serological tumor marker in clinical practice. Monitoring the activity level of NSE is an important method to evaluate the progression of the disease, the effect of treatment and predict the recurrence. The method is earlier and more convenient than an X-ray examination. Antigen–antibody binding is mainly used in the current photoelectrochemical sensors. Li et al. used dopamine as a sensitizer to modify a photoelectrochemical immunosensor on hyperbranched TiO_2_ arrays, which can detect NSE in serum with good selectivity, stability and reproducibility [31]. The main advantages take advantage of the high selectivity of antibody–antigen-specific interactions and the signal amplification strategy of dopamine-sensitized PEC sensors. Dopamine-sensitized titania can shorten the carrier diffusion distance and enhance the light-harvesting efficiency and charge-collection efficiency, thereby improving the performance of the obtained PEC sensor. Second, this PEC immunosensor can detect analytes (target compounds) based on changes in the photocurrent during the immune response. The incubation time and temperature of the antigen–antibody interaction have important effects on the performance of the immunosensor. The experiments showed that both the incubation temperature and the pH value of PBS have important effects on the performance of the sensor. Among them, the optimal incubation temperature was 35 °C, and the optimal pH = 7.4; at 20–35 °C, the photocurrent increased with the increase of the incubation temperature, which was due to the immune reaction between NSE and anti-NSE. However, when the temperature exceeds 35 °C, the photocurrent response decreases, which can be explained by the irreversible variability of NSE caused by high temperatures. Additionally, this sensor has good specificity, stability and repeatability. When the sensor is used for the detection of NSE, it has a good linear relationship in the range of 0.1–1000 ng/mL, and the detection limit is 0.05 ng/mL. Comparatively, Zhang et al.’s photoelectrochemical immunosensor based on a Z-scheme WO_3_/NiCo_2_O_4_ nanoarray p-n heterojunction utilizes the LSPR effect of Au to convert thermions into a photocurrent to achieve signal amplification [33]. As a representative spinel-type binary metal oxide, NiCo_2_O_4_ has good electrical conductivity, and WO_3_/NiCo_2_O_4_ with a large specific surface area provides a large number of active centers for the loading of polydopamine (PDA) films. The good electrical conductivity and good biocompatibility of PDA films also provide a good foundation for the design of PEC immunosensors. Similarly, in the range of 0.1 pg/mL–50 ng/mL, the logarithmic value of the NSE concentration has a linear relationship with the photocurrent intensity, and the detection limit was 0.07 pg/mL. Moreover, it has good stability, repeatability and selectivity. Other tumor markers in serum, such as SCCA, CEA, etc., will not affect the experimental results, indicating their specificity and selectivity.

#### 4.1.4. NSE Detection Based on Piezoelectric Biosensors

NSE, a cell-specific isoenzyme of the glycolytic enzyme enolase, is a crucial protein in the human brain [34] The increase of NSE is shown in patients who experience brain damage. To better detect the content of NSE, Elisabeth et al. utilized the quartz crystal microbalance (QCM) biosensor A100 to do research on the epitope characterization of NSE [35]. Accordingly, carboxyl-coated chip surfaces were firstly covalently fixed by polyclonal rabbit anti-mouse immunoglobulin antibodies. Then, a mixture of monoclonal antibodies of IgG1, IgG2a, IgG2b, IgG3 and other isoforms unrelated in mice was added to saturate the captured surface. Statistically, a positive result meant a frequency shift over 10 Hz at antibody binding. Combined with the data of SPR, QCM, cross-inhibition and immunoassay construction, antibodies could be classified into five epitope groups (A–E). This grouping has a high degree of consistency among different methodologies.

#### 4.1.5. NSE Detection Based on Aptasensors

NSE plays an important role in the screening, early diagnosis, efficacy evaluation and prognosis judgment of small cell lung cancer. Zheng et al. used subtractive SELEX to design DNA aptamers with high affinity and selectivity for NSE [36]. In the secondary structure prediction of DNA aptamers, the authors found that the stem–loop structure appeared more frequently in the secondary structure of these aptamers, so three aptamers were selected to form a stable B-form, stem–loop conformation. Compared with antibodies, aptamers have the advantages of high affinity, small molecular weight, good repeatability and good stability. In the optimization of the aptasensor, it was found that, when the concentration of Apt-5 was between 25 and 100 pmol, the luminescence intensity gradually increased with the increase of the doping amount of Apt-5. In the control experiment, it was observed that the chemiluminescence intensity of normal serum and non-small cell lung cancer serum was close to blank, while the luminescence intensity increased significantly when combined with NSE-positive serum. It can be explained that it has a certain specificity and high selectivity. When the NSE concentration was in the range of 1–100 ng/mL, there was a linear relationship between the luminescence intensity and the concentration of the novel Apt-5 sensor. The detection limit of NSE is 0.1 ng/mL, which is 2.5 times lower than the 0.25 ng/mL limit of ELISA used in hospitals.

### 4.2. CEA Detection

CEA is a tumor-associated antigen firstly extracted from colon cancer and embryonic tissues, which is formed in the cytoplasm and then secreted out of the cell and into the surrounding body fluid. In the past, CEA has been used as an early tumor marker of colon cancer and rectal cancer. Increased CEA is also found in the serum of breast cancer, lung cancer and other malignant tumors.

#### 4.2.1. CEA Detection Based on Electrochemical Biosensors

**Figure 6:**
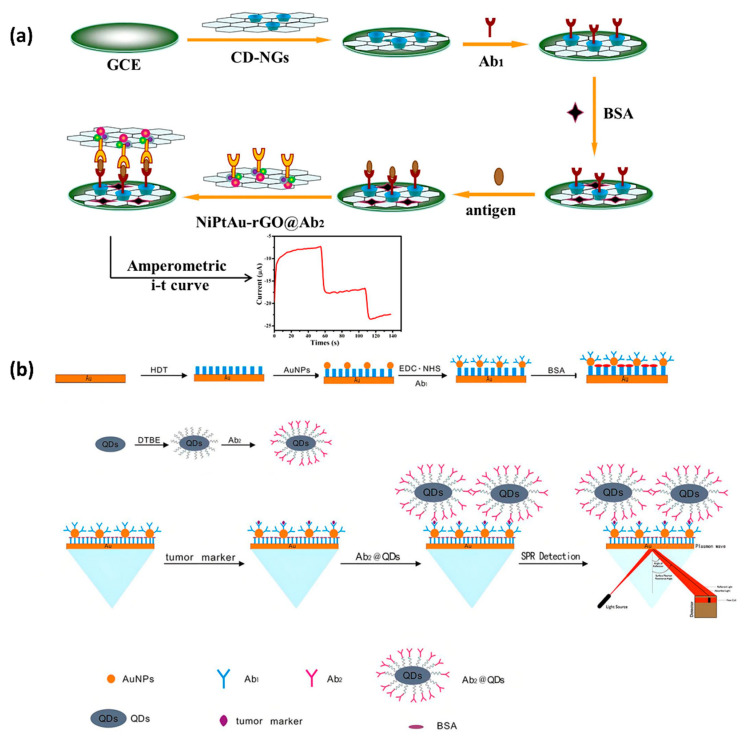
(**a**) The schematic illustration of the fabrication process of the sandwich-type electrochemical immunosensor. Reprinted with permission from [39]. (**b**) The detection procedure. Reprinted with permission from [40].

In recent years, electrochemical immunosensors have been widely used in biological monitoring due to their advantages of high sensitivity, fast detection speed, miniaturization and low cost. In the past, in order to improve the sensitivity and selectivity of biosensors, many methods have been used to amplify the signal of the immunosensor; among which, the most popular method is to use functional materials with excellent catalytic performance. For example, Tian et al. designed a novel ultrasensitive sandwich electrochemical immunosensor for the quantitative detection of CEA, as shown in Figure 6a [39]. CEA is known to be a standard tumor marker for gastrointestinal tumors. The electrochemical sandwich immunosensor has good stability, accuracy and selectivity for the detection of CEA. In addition, the detection limit of the immunosensor was merely shallow, 0.27 pg/mL, which was conducive to the early diagnosis and follow-up treatment of patients with colon cancer and gastric cancer.

#### 4.2.2. CEA Detection Based on Optical Biosensors

To provide a signal amplification strategy, Wang et al. used antibody–quantum dot (QD) conjugates to detect CEA in a sensitive and quantitative way (Figure 6b). After AuNP@Ab1 conjugates captured the target and Ab2@QD conjugates, the signal changes of the SPR biosensor increased due to the mass enhancement of the quantum dots [40]. In addition, Liu et al. coupled single-domain anti-CEA antibodies (sdAbs) to the surface of the sensor to improve the biosensing activity of SPR biosensors [41]. To further improve biosensors, Al-Enezi et al. developed a novel technique using Affimer-based Eu^3+^ complexes as nanobiosensors for the optical biosensing of CEA. This method has high sensitivity, with a detection limit below 100 fM, especially in clinical trials as a targeting strategy [42].

Li, Shi, Sun, Li and Liu (2016) developed a biosensor based on the fluorescence energy transfer (FRET) between upconverting nanoparticles (UCPs) and palladium nanoparticles (PdNPs) for the detection of CEA. The coordination interaction between the CEA aptamer and PdNPs brought UCPs and PdNPs in close proximity, causing fluorescence quenching. However, when the CEA was introduced, the binding of CEA to the aptamer weakened the above-mentioned coordination, so that the recovery of fluorescence could be detected to quantify the concentration of CEA [43].

Similarly, Yu, Zha, Tang, Qiu and Liu (2022) synthesized polydopamine-coated upconversion nanoparticles (UCNPs@PDA) and CEA aptamer-functionalized AuNPs (AuNP–CEA aptamer) to form a new modification-free fluorescent biosensor. The interaction between the AuNP–CEA aptamer and UCNPs@PDA resulted in the process of fluorescence resonance energy transfer from the latter to the former. The strong affinity of CEA with its aptamer led to the separation of the AuNP–CEA aptamer and UCNPs@PDA, so the recovery of the fluorescence could indicate the change in CEA concentration. This biosensor provided a linear range from 0.1 to 100 ng/mL with a detection limit of 0.031 ng/mL in aqueous solution and 0.055 ng/mL in human serum [44].

#### 4.2.3. CEA Detection Based on Photoelectrochemical Biosensors

Wang et al. (2016) developed a label-free photoelectrochemical immunosensor for the detection of CEA. They prepared two-dimensional TiO_2_ nanosheets were modified with carboxylated graphitic carbon nitride (g-C_3_N_4_), which had a strong photocurrent. Then, the antibody of CEA was bound to the nanosheets, and the specific binding of CEA and its antibody resulted in a decrease in the photocurrent [45].

Nie, Tang, Zhang, Wang and Guo (2018) developed a kind of label-free photoelectrochemical immunosensor that had good stability and specificity. They introduced electrochemically reduced graphene oxide into poly(5-formylindole) to prepare a nanocomposite that could generate a high photocurrent and modified the antibody on the surface of the electrode to reduce the photocurrent through the specific binding of CEA and its antibody. It had a low detection limit of 0.14 pg/mL [46].

#### 4.2.4. CEA Detection Based on Piezoelectric Biosensors

CEA is a kind of tumor marker associated with several specific cancers or carcinomas. Its normal concentration in a healthy adult is less than 5 ng/mL [47]. The changes in CEA can be used to measure the treatment of various cancers. Therefore, it is necessary to establish an effective method to detect CEA at low concentrations in the clinical diagnosis. Accordingly, Zhang et al. designed a novel multiarray immunoassay device based on a plug-in model of piezoelectric (Pz) immunosensor to detect the quantity of CEA [48]. The device utilized a 2 × 5 model Pz multiarray immunoanalyzer assembled with a plug-in Pz sensor, which can simultaneously detect multiple tumor markers. As a result, the speed of detection was eight times faster than a single immunosensor, and the detection limitation of CEA ranged from 1.5 μg/mL to 30 μg/mL. On the one hand, the advantages of Pz devices are their portability, simplicity, low costs and suitability in the real-time monitoring of bio-specific interactions with high sensitivity and specificity. On the other hand, disadvantages such as the difficulty in liquid-phase oscillation and stability limit its application.

#### 4.2.5. CEA Detection Based on Aptasensors

Recently, various aptasensor bioanalytical methods for CEA have been reported, such as the fluorescence and electrochemical methods. Bai et al. designed an aptasensor platform based on a hybridization chain reaction (HCR) and G-quadruplex DNAzyme for the fluorescence detection of CEA. This method needs the labeling or modification of the oligonucleotide, which combines the specific recognition property of the aptamer with the quadratic signal amplification strategy of the HCR and G-quadruplex DNAzyme. In addition, the fluorescent aptasensor shows a detection limit of 0.5 nM and a linear relationship ranging from 0.25 to 1.5 nM toward the CEA. Without being affected by interfering proteins such as IgG, AFP and PSA, this aptasensor has a high selectivity to CEA and is successfully applied to the CEA analysis in diluted human serum samples [49].

Zhai et al. reported a DNAzyme-catalyzed label-free aptasensor based on a multifunctional dendrimer-like DNA nanoassembly. Through an amplified and label-free differential pulse voltammetry (DPV) electrochemical signal, the peak current of this aptasensor correlated linearly with the CEA concentration, with a linear range of 2–45 ng/mL and a LOD value of 0.24 ng/mL. The aptasensor also showed high specificity and reproducibility [50].

### 4.3. PSA Detection

PSA is a 32-kDa single-chain glycoprotein belonging to the serine protease family with tissue specificity and chymotrypsin-like effects. It can be synthesized in normal and cancer-like epithelial cells. Typically, the PSA levels in the blood are usually very low, but most of the men with prostate cancer have high levels. According to the survey, prostate cancer is the second-most common cancer among men worldwide. In recent years, the application of prostate-specific antigen detection technology has promoted the improvement of the diagnosis and treatment of prostate cancer. However, previous methods based on immunological recognition, such as colorimetry and electrochemiluminescence, are complex in preparation and operation, which are difficult to apply to the grassroots [51].

#### 4.3.1. PSA Detection Based on Electrochemical Biosensors

**Figure 7:**
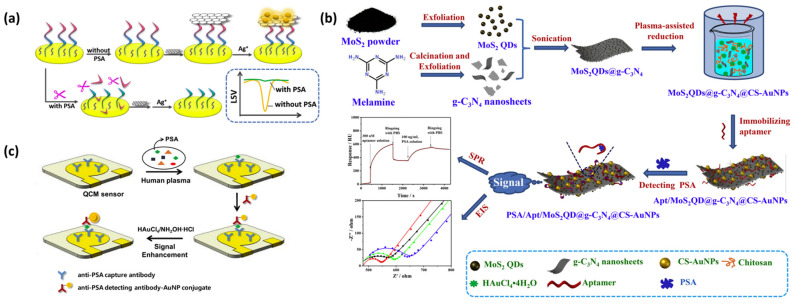
(**a**) Schematic diagram of detecting PSA by an electrochemical biosensor based on peptide cleavage. Reprinted with permission from [52]. (**b**) Schematic diagram of the PSA test. Reprinted with permission from [55]. (**c**) Schematic of the sandwich immunoassay format in the QCM sensor chip combine with gold-staining signal amplification. Reprinted with permission from [61].

Accordingly, Meng et al. demonstrated a novel electrochemical biosensor for the prostate-specific antigen (PSA) sensitivity analysis. The electrochemical method based on peptide cleavage and the detailed sensing principle is shown in Figure 7a. The detection and analysis performance, selection performance and clinical analysis of the sensor achieved satisfactory results. In this study, the disadvantages of the complex production of the traditional methods were improved, and the PSA analysis method was supplemented, providing a good attempt for the detection of PSA [52].

#### 4.3.2. PSA Detection Based on Optical Biosensors

In order to obtain higher PEC (photoelectrochemical) efficiency, Zhang et al. developed a new type of label-free photoelectrochemical immunosensor based on SnS_2_@mpg-C_3_N_4_ to detect PSA [53]. The linear detection range of the photoelectrochemical immunosensor was 50 fg/mL–10 ng/mL, and the detection limit was 21 fg/mL with high sensitivity. Kong et al. developed a labeling-free fluorescent sensor based on the AIE-silica nanosphere (SiO_2_ NP) for PSA detection [54], which illustrated high sensitivity and selectivity for PSA with a detection limit of 0.5 ng/mL. In addition, Duan et al. designed a nanoparticle (denoted as MoS_2_QDs@g-C_3_N_4_@CS-AuNPs) to construct an SPR sensor based on surface plasmon resonance (Figure 7b). Aptasensor based on MoS_2_QDs@g-C_3_N_4_@CS-AuNPs presented a strong bio-binding affinity for PSA, with a rapid response, highly sensitive detection limit of 0.72 ng/mL and wide detection range of 1.0–250 ng/mL PSA concentration [55].

Pei et al. (2015) developed a fluorescent turn-on nanoprobe to detect PSA based on graphene oxide quantum dots@silver (GQDs@Ag) core–shell nanocrystals. One probe was assembled by quantities of GQDs so that the fluorescent signal could be significantly enhanced. Using magnetic beads (MBs) immobilized with anti-PSA antibody as a separable capture probe and GQDs@Ag as a detection probe, PSA was detected by the sandwich method. The biosensor showed a detection limit of 0.3 pg/mL and the linear range from 1 pg/mL to 20 ng/mL [56].

Yang et al. (2018) developed a fluorescent biosensor based on peptide/Fe_3_O_4_@SiO_2_-Au nanocomposites (MNCPs). 5-FAM-tagged peptides were self-organized on the surface of MCNPs to help quench the fluorescence. After PSA antigen specifically recognized and cleaved the peptides, the fluorescence was then recovered. The biosensor had a wide range of concentrations of PSA, from 1 × 10^−12^ g/mL to 1 × 10^−9^ g/mL, with a detection limit of 3 × 10^−13^ g/mL [57].

#### 4.3.3. PSA Detection Based on Photoelectrochemical Biosensors

Zhu et al. designed a sensitive novel label-free photoelectrochemical immunosensor, which used an Ag_2_S-sensitized Ag/AgBr/BiOBr heterojunction to improve the photocurrent response and sensitivity. The limit of detection of the photoelectrochemical immunosensor for PSA was 0.25 pg/mL, and the linear range was from 0.001 to 50 ng/mL. The photocurrent of this immunosensor reduced linearly with the logarithm of the PSA concentration, with the advantages of a wide linear range, good stability, high reproducibility, low cost and good selectivity [58].

Zhao et al. developed a peptide-based photoelectrochemical (PEC) biosensor that was constructed based on the CdTe/TiO_2_-sensitized structure as the electrode and CuS nanocrystals as a signal amplifier. The PEC biosensor was constructed based on the reaction that the prostate-specific antigen (PSA) was capable of cleaving a specific amino acid sequence, so that it revealed good specificity, stability and reproducibility, with a linear range from 0.005 to 20 ng/mL and a LOD value of 0.0015 ng/mL. This biosensor shows the potential applications of the photoelectrochemical biosensor in bioanalysis, disease diagnostics and clinical biomedicine [59].

#### 4.3.4. PSA Detection Based on Piezoelectric Biosensors

QCM is a highly sensitive sensor device that can measure the mass loading effect by using the resonant frequency that varies with the mass of a given sensing surface [60]. Jiwon Kwak et al. showed that gold nanoparticles were used for signal amplification and designed highly sensitive piezoelectric immunosensors. The QCM sensing system used in the above research included a detection module and a fluid module to obtain the real-time response of the sensor during the immunoanalysis process. The experimental results showed that the QCM-based sensor with gold holding enhancement on the surface could improve the sensitivity of immune detection. The limit of detection (LOD) of the PSA immunoassay in gold-enhanced human serum was 48 pg/mL, and in the absence of signal amplification, the detection limit was 687 pg/mL. In addition, Jiwon Kwak et al. showed in another study that, when using a quartz crystal microbalance (QCM) biosensor to measure the PSA concentration in human plasma, the sensitivity and reproducibility were improved by a factor of two and three, respectively (Figure 7c,d) [61]. This is because human plasma contains a large amount of fibrin, which can affect the experimental results. The role of the sulfate protein is to combine fibrin to form precipitation, which can be easily removed, thus improving the detection sensitivity. In the above study, the limit of detection (LOD) of PSA in human plasma was 112 pg/mL, which was lower than the clinical threshold.

#### 4.3.5. PSA Detection Based on Aptasensors

Tang et al. developed a visible and near-infrared light dual responsive photoelectrochemical aptasensor based on MoS_2_ nanoflowers and gold nanobipyramids, the method calibration to determine the PSA through the “signal-off” and “signal-on“ models. The limit of detection for PSA in the “signal-off” or “signal-on” mode is calculated to be 1.75 pg/mL and 0.39 pg/mL, respectively. The dual-responsive photoelectrochemical aptasensor showed a linear response to the logarithm of the PSA concentration in the range of 0.005–100 ng/mL under the optimized conditions, which was also employed for determining the PSA in clinical serum samples with satisfactory selectivity and excellent accuracy [62].

Wan et al. designed a new sandwich electrochemical aptasensor with a signal amplification strategy by coupling tyrosinase (Tyr)-triggered redox cycling with nanoscale porous carbon (NCZIF) to improve the efficiency of the aptasensors. The LOD value of the proposed electrochemical aptasensor for PSA was 0.01 ng/mL, and the linear range was from 0.01 to 50 ng/mL. The method demonstrates simple, rapid, reliable, great performances and has potential value in practical applications [63].

### 4.4. SCCA Detection

#### 4.4.1. SCCA Detection Based on Electrochemical Biosensors

The squamous cell carcinoma antigen (SCCA) has been considered as an effective tumor marker in the diagnosis of cervical cancer, especially in the evaluation of the occurrence and development of cancer, treatment effect and monitoring prognosis recurrence. Different from the traditional fluorescence immunoassay and chemiluminescence immunoassay, Liu et al. designed a novel ultrasensitive sandwich electrochemical immunosensor for the quantitative detection of SCCA in consideration of high biological specificity. After optimization, under the optimal conditions of PBS pH = 7.0, CD-GN 1.0 mg/mL and Pt/PdCu-3DGF 1.5 mg/mL, the immunosensor had good reproducibility and stability, relatively comprehensive linear range and lower detection limit. This sensor provides a new possibility for SCCA detection and has broad application prospects [64].

**Figure 8:**
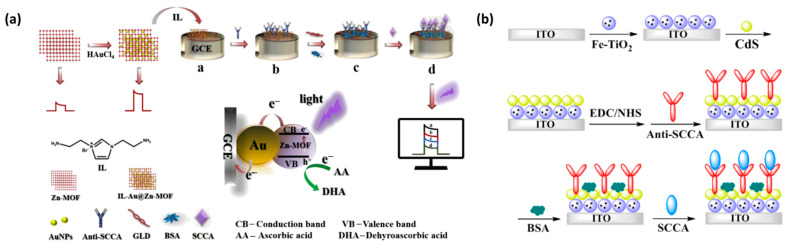
(**a**) The overall fabrication process of the PEC immunosensor and the specific response to SCCA, and the mechanism for the photocurrent generation. Reprinted with permission from [65]. (**b**) Schematic diagram of the PEC immunosensor. Reprinted with permission from [67].

However, in practice, the concentration of SCCA in the serum of healthy adults is less than 1.5 ng/mL, which is a great challenge for the selection and accurate determination of SCCA. In order to overcome the disadvantages of the complex operation and high cost of the previous detection methods, Qiu et al. made a PEC immunosensor platform based on Au-NPs@Zn-MOF nanocomposite materials (Figure 8a). Compared with the previously reported PEC immunosensor, this experiment confirmed that the Au-NPs@Zn-MOF-based PEC immunosensor had the characteristics of high selectivity, high sensitivity and high stability for SCCA detection. However, the preparation of gold nanocomposites is still complex, time-consuming and labor-intensive. Hence, the immunosensor still had significant challenges and room for improvement in the early detection of tumor markers [65].

#### 4.4.2. SCCA Detection Based on Optical Biosensors

SCCA is a 48-kDa glycoprotein, which is a subtype of tumor-associated antigen, TA-4. It is found in the cytoplasm of squamous cell carcinomas of the uterus, cervix, lung and head and neck. It is the earliest tumor marker used in the diagnosis of squamous cell carcinoma and can be used as an auxiliary diagnostic index and prognostic monitoring indicator for a few cancers. For example, Qian et al. developed an LSPR biosensor based on the triangular silver nanoparticles array with monoclonal anti-SCCA antibodies immobilized on the chip to directly detect SCCA [66]. Its analytical performance is superior to the routine CLIA method. In addition, Fan et al. developed a novel label-free immunosensor platform based on CdS-sensitized Fe-TiO_2_ nanocomposites for the ultrasensitive detection of SCCA (Figure 8b). Due to the good PEC response of CdS-enhanced Fe-TiO_2_, the biosensor had a wide detection range from 0.001 ng/mL to 75 ng/mL and a low detection limit of 0.22 pg/mL [67]. To create a more efficient photoelectrochemical biosensor, Wei et al. designed a new photoelectrochemical immunosensor based on Au-NPs@Zn-MOF for SCCA detection with high sensitivity, high selectivity, stability and repeatability [65].

#### 4.4.3. SCCA Detection Based on Photoelectrochemical Sensors

Fan et al. (2019) developed a visible light photoelectrochemical immunosensor based on BiOBr/Bi_2_O_3_ heterostructures for the ultrasensitive detection of SCCA. By the self-sacrificial synthesis method, BiOBr interacted with S2 ions to form Bi_2_S_3_ on the surface of BiOBr microspheres, which had excellent visible light photoelectrochemical activity. Dopamine is self-polymerized to form a polydopamine film on its surface to further consolidate photoelectric signal stability and promote antibody binding. The intensity decreased linearly with the logarithm of the SCCA concentration in the range of 0.001–75 ng/mL with a detection limit of 0.3 pg/mL [68].

### 4.5. CA19-9 Detection

CA19-9 is a tumor marker of pancreatic cancer, gastric cancer, colon cancer and gallbladder cancer. Although CA19-9 is mainly used for the differential diagnosis and condition monitoring of pancreatic cancer, its positive rate is also high in colorectal cancer.

#### 4.5.1. CA19-9 Detection Based on Electrochemical Biosensors

**Figure 9:**
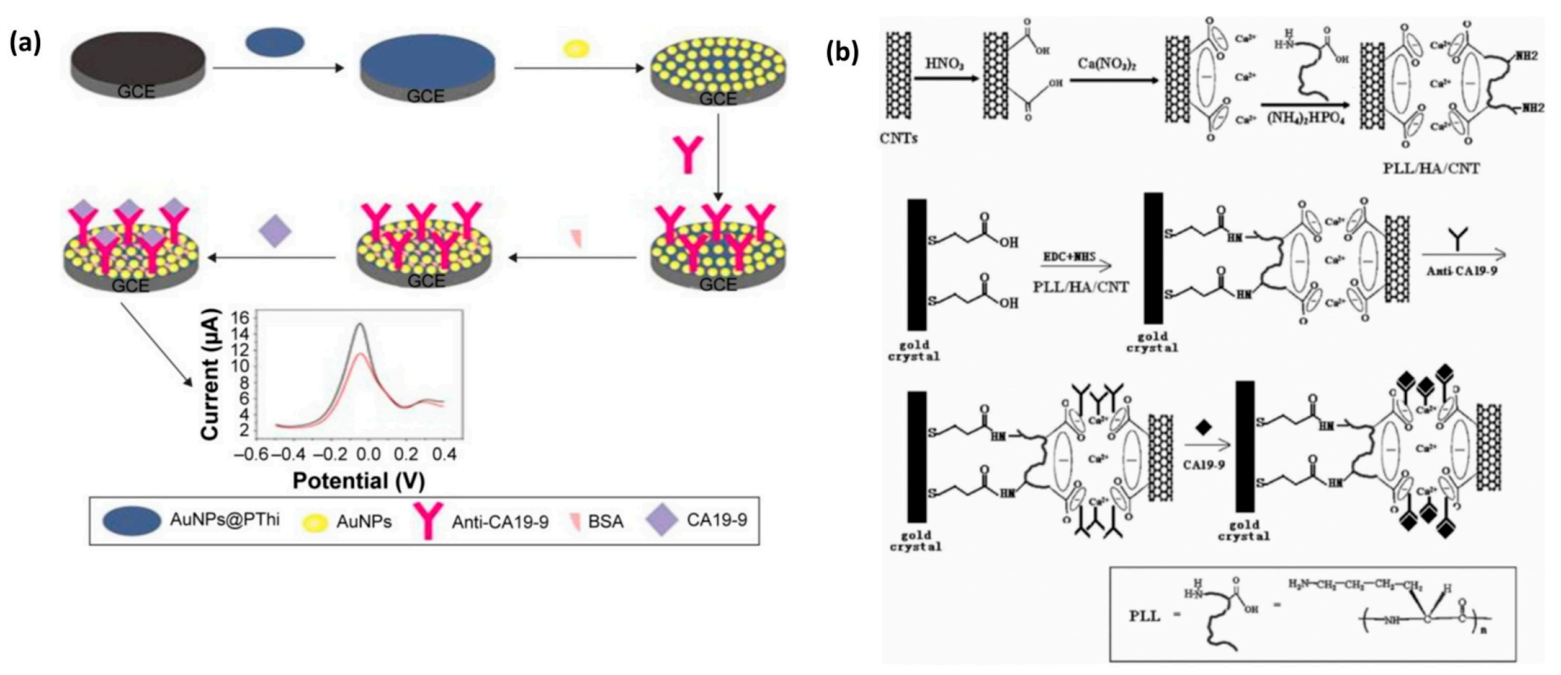
(**a**) Schematic diagram of the electrochemical immunosensor. Reprinted with permission from [69]. (**b**) Schematic diagram of a synthetic immunosensor made of PLL/HA/CNT hybrid materials. Reprinted with permission from [73].

The detection of CA19-9 based on electrochemical biosensors has been extensively studied. For example, Huang et al. used a polysulfide–gold composite (AuNPs@PThi) as a probe to fabricate a new electrochemical immunosensor (Figure 9a). Under optimal conditions, the linear range of the electrochemical immunosensor was estimated to be 6.5–520 U/mL, the detection limit was 0.26 U/mL and the signal-to-noise ratio was 3. The experimental verification of the characterization proves that it is not only beneficial to signal amplification but also sensitive, stable and reliable to detect CA19-9 [69]. In addition, Mo et al. designed a novel electrochemical luminescence (ECL) immunosensor based on spatially resolved biopotential technology, which could also be used to detect CA19-9. The detection linear range of CA 19-9 was 0.0001–10 U/mL, with a detection limit of 31 μU/mL. This new sensor presents a lower detection limit and a wider linear range, which provides a new avenue for the application of the sensor in the clinical field [70].

#### 4.5.2. CA19-9 Detection Based on Optical Biosensors

Based on SERS technology, Zhou et al. developed a sandwich structure composed of nano-Si immune probes and a SiC@Ag SERS active immune substrate as an ultrasensitive immunoassay optical biosensor for detecting tumor markers in human serum [71]. Strong SERS “hot spots” between silver nanoparticles in the structure led to obvious Raman enhancement. However, due to the purchase of reagents, the maximum limit of the antigen could not be determined in the case of a high concentration. Hence, the existence of false negatives needs to be explored. Although this method still has many unsolved problems, it still has potential application value in the clinical diagnosis.

#### 4.5.3. CA19-9 Detection Based on Photoelectrochemical Sensors

Wang et al. invented a photoelectrochemical sensor, LF-LAPECS. He can sensitively and quickly detect multiple tumor markers on the electrode, especially CA19-9, and can be applied to clinical serum samples through experiments. The calibration range of this invention for CA19-9 is 0.1–1000 U/mL, and the detection limit is 0.01 U/mL, which shows that it has a wide calibration range and a low detection limit and has a high potential for clinical detection [72].

#### 4.5.4. CA19-9 Detection Based on Piezoelectric Biosensors

Compared with the first two biosensors, there is relatively little research on the detection of CA19-9 by piezoelectric sensors. Huang et al. fabricated an improved QCM immunosensor using poly-L-lysine/hydroxylapatite/carbon nanotube (PLL/HA/CNT) hybrid particles (Figure 9b), which are able to detect CA19-9 serum in the dynamic concentration range of about 12.5–270.0 U/mL. This method is not limited by the shape of the sensor to a large extent and provides a new idea for the clinical detection of CA19-9 [73].

### 4.6. TP53 Detection

The TP53 tumor suppressor has a strong effect on the growth regulation, genetic stability and proliferation control of cells. Mutations in the TP53 gene, which occurs in almost 50% of human tumors, are associated with the accumulation of the mutant protein in the nucleus of tumor cells, resulting in increased concentrations in extracellular fluids, such as blood, urine and saliva [74].

#### 4.6.1. TP53 Detection Based on Electrochemical Biosensors

Elif et al. developed an electrochemical immunosensor for the sensitive detection of TP53 based on an ITO electrode coated with a chitosan/carbon black composite (chitosan–CB) layer. Compared with traditional sensors, the newly prepared immunosensor demonstrates good sensitivity, stability and repeatability due to the affinity reaction on the electrode. In addition, this unlabeled immunosensor responds rapidly to TP53 antigen and, thus, has long-term stability and high selectivity, and can also be used for the determination of TP53 in serum samples. In their study, the sensor had a wide linear range of 0.01–2 pg/mL and a low detection limit of 3 fg/mL. These advantages make it a great advantage in the field of clinical detection [75].

**Figure 10:**
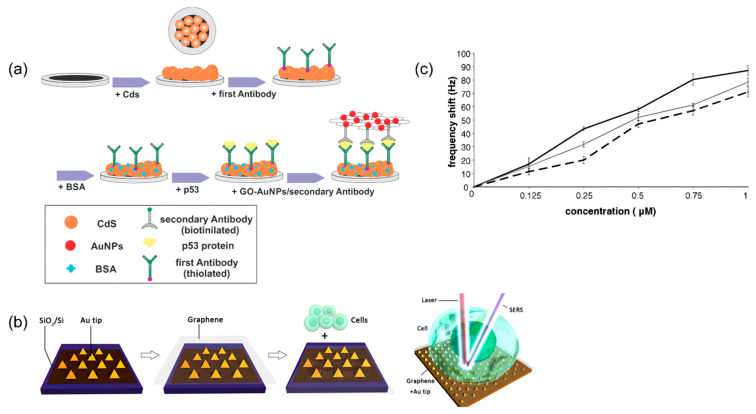
(**a**) Schematic diagram of the ECL immunosensor based on CdS NCs and tGO-AuNPs. Reprinted with permission from [76]. (**b**) Schematic diagram of the Gr-Au tip platform for SERS detection, and a pattern diagram of cancer cells deposited on the Gr-Au tip platform. Reprinted with permission from [77]. (**c**) The relative calibration curves of different concentrations of fully complementary and mismatched oligonucleotides. Reprinted with permission from [81].

To further improve the sensitivity, Reza et al. invented a new electrochemical luminescence (ECL) immunosensor (Figure 10a) to selectively quantify the TP53 protein, which utilized the principle of AuNP-enhanced ECL emission from CdS nanocrystals (CdS NCS). Under optimal conditions, the linear range of the ECL immunosensor was between 20 and 1000 fg/mL, and the calculated detection limit was 4 fg/mL. Although the detection limit is slightly higher than the previously introduced sensors, it still has great potential for clinical cancer detection due to its high sensitivity [76].

#### 4.6.2. TP53 Detection Based on Optical Biosensors

P53 is an important tumor marker of colon cancer and plays an important role in diagnosing colon cancer. SERS can be used to identify TP53 −/− (gene knockout) and TP53 +/+ (wild type). In addition, SERS can identify three different states of the cells (burst, alive and dead), because there are unique fingerprints in the three different cellular states that can be used to distinguish them. The experiments were performed on the above cells using a label-free graphics/gold nanopyramid-based SERS platform and principal component analysis (PCA), distinguishing between TP53 −/− (gene knockout) and TP53 +/+ (wild type) and three types of cells (Figure 10b) [77]. However, the technology is still in the experimental stage, and the clinical samples are insufficient. In addition, how to prevent the influence of patient substances on the SERS signal is also a key issue. Nevertheless, the technique has great potential in cancer cell differentiation.

Xu et al. (2018) developed a sensitive fluorescent biosensor based on DNA-functionalized Fe_3_O_4_ nanoparticles. The consensus DNA, which was immobilized on aminated-dextran modified Fe_3_O_4_ NPs, was tagged by Cy-5 to generate fluorescent signals, and the interaction between DNA and the p53 protein can lead to the decrease of fluorescent emission. This kind of biosensor had a detection limit of 8 pM, with the linear range from 50 pM to 2 nM [78].

#### 4.6.3. TP53 Detection Based on Photoelectrochemical Biosensors

The P53 gene is the most mutated gene in human cancer [79]. The disadvantage of the current photoelectrochemical sensor is that it can only realize the analysis of a single detection object, which is inefficient. While Zheng et al. successfully synthesized wavelength-selective photoactive materials and constructed a new type of PEC biosensor for the detection of multiple analytes on a single interface, Zheng’s experiments successfully used photoactive materials. MPT NPs and TiO_2_ NPs are the ORVOA 1 gene and p53 gene as signal indicators, respectively, which can realize the simultaneous detection of the two genes [80]. Among them, in the preparation of TiO2 NPs/AuNPs/hairpin DNA 2/HT photoactive bionanoconjugates (probe 2), improvements were made to synthesize TiO_2_ nanoparticles with spherical flower-like nanostructures, and an enzyme-assisted target recovery amplification strategy was also introduced to provide efficient signal amplification by cleaving the signal probe upon binding to the target, thereby releasing and reusing the target for ultrasensitive monitoring. By hybridizing to DNA captured on the electrode surface, the cleaved probe can be immobilized on the electrode, resulting in a photocurrent response for the quantitative determination of the target. The experimental results showed that the presence of the p53 gene (2.5 fM) caused the excitation light source to respond to a photocurrent of 106.4 nA at 365 nm, while the photocurrent response to a 590-nm excitation light source did not change, while the ORVOA 1 gene had the opposite. The photocurrent of the p53 gene increased linearly with the increase of concentration in the range of 25 aM~2.5 pM. When the interfering substance is 100 times that of p53, it has no effect on the photocurrent, which can prove its good selectivity and specificity.

#### 4.6.4. TP53 Detection Based on Piezoelectric Biosensors

The detection of the p53 point mutation (codon 248) in PCR-amplified samples was investigated by Dany et al. using the same transduction principle as 9.5 MHz quartz crystals [81]. They successfully immobilized the p53-biotinylated probe on the sensor surface with 9.5 MHz AT-Cut quartz crystals (14 mm) decorated with gold plating (42.6 mm^2^ area) on both sides. As a result, the experimental threshold was 0.12 M for all the tested samples (Figure 10c). Furthermore, the highest signal difference and best discrimination concentration among the three oligonucleotides was 0.25 M.

### 4.7. Other Tumor Biomarkers Detection

ATPase H+ Transporting Accessory Protein 1 (ATP6AP1), which is a component of a multi-subunit enzyme in Vacuole ATPase (V-ATPase), is highly expressed in breast cancer tissues [82].

#### 4.7.1. ATP6AP1 Detection Based on Piezoelectric Biosensors

**Figure 11:**
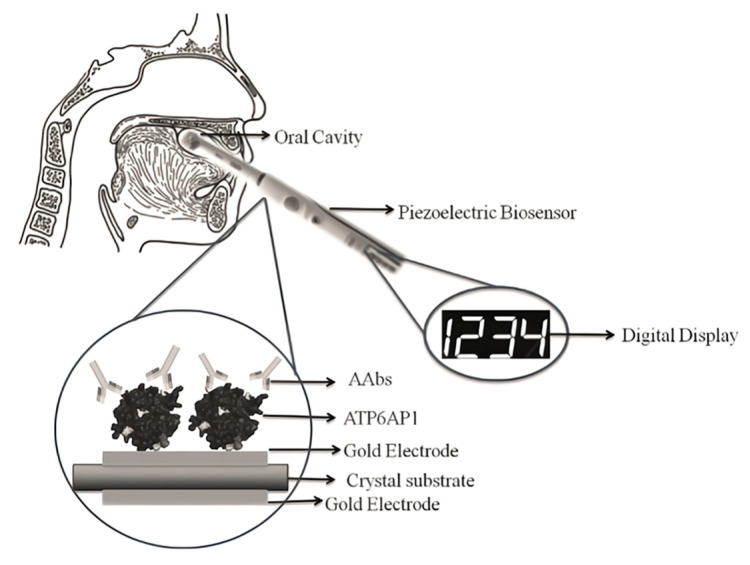
Utilizing the salivary autoantibodies against ATP6AP1 to detect breast cancer through the biosensor. Reprinted with permission from [84].

Breast cancer is a common malignant disease among women [83], accounting for 7–10% of all cancers in China. The cause of breast cancer is unknown. According to the current studies, the related factors of breast cancer include age of menarche, age of early menopause, fertility, breastfeeding, drinking, obesity, etc. [84]. Breast cancer cells are mostly derived from mammary ducts and mammary lobules, which can be divided into noninfiltrating cancer, invasive special cancer and invasive nonspecial cancer. Currently, the methods of diagnosing breast cancer include tissue biopsy and mammography. Tissue biopsy is the “gold standard” for disease diagnosis. Mammography is also widely used in hospitals for diagnosing breast cancer. However, the major limitation of breast mammography is that only 70% of breast cancers can be detected. This is because tumors smaller than 7.5 mm are difficult to be found. Interestingly, Sania et al. [84] found that ATP6AP1 autoantibodies (AAb) are produced in cancer patients, with the highest concentration in breast cancer patients. Therefore, it can be used to provide a new idea for the early detection of breast cancer. ATP6AP1 autoantibodies (AAb) are induced and secreted in the serum of patients due to changes in the proteoglycan expression or structure. The concentration of the ATP6AP1 autoantibody could be detected by the quartz crystal microbalance. The strategy was to overlay the ATP6AP1 autoantibody on the surface of the crystal where the antigen and antibody bind. The biosensor can convert the vibration frequency into an electrical signal and be observed directly in the form of a digital signal (Figure 11). However, ATP6AP1 autoantibodies (AAb) also have the disadvantages of low sensitivity (30%) and high specificity (95%) in early detection.

#### 4.7.2. CA15-3 Detection Based on Electrochemical Biosensor

The tumor marker CA15-3, a serum-based product of the MUC1 gene, is overregulated in breast cancer and is also overexpressed in many other types of cancer, including lung, ovarian, pancreatic and colon cancers. Therefore, the detection of CA15-3 is particularly important [85]. Hong et al. used cyclic voltammetry to monitor changes in the biosensor current signals to detect CA15-3. Ferrocene carboxylic acid (FC-COOH)-doped silicon microspheres (SNPs) were modified on the surfaces of gold electrodes and could interact with CA15-3 antibody-conjugated glutaraldehyde linkage. The linear range of the method was 2.0~240 U/mL, and the detection limit was 0.64 U/mL. Han et al. also constructed an electrochemical biosensor using PEDOT and peptides to detect CA15-3. The device modifies polypeptide-doped PEDOT onto the electrode surface, which can detect CA15-3 in serum without being affected by biofouling and has high sensitivity and long-term stability. The linear range is 0.01~1000 U/mL, and the detection limit is 3.34 U/mL [86].

#### 4.7.3. HER2 Detection Based on Photoelectrochemical Sensor

HER2 belongs to the receptor tyrosine kinase family, which plays an important role in promoting the biological behavior of breast cancer cells, such as division, proliferation and migration. HER2-positive breast cancer accounts for 20–30% of the molecular types of breast cancer [87]. The characteristics of HER2-positive breast cancer are a high degree of malignancy and poor prognosis [88]. The most common tumor biology is HER2-negative luminal tumors (approximately 70%) [89]. Luo et al. prepared a sensitive photoelectrochemical (PEC) sensor for the detection of human epidermal growth factor receptor 2 (HER2), using a hexagonal carbon nitride tube (HCNT) as the photoactive material. Magnetic Fe_3_O_4_ nanospheres (MNs) modified with anti-HER2 antibodies efficiently capture HER2 in serum samples. MNs are spherical and relatively uniform in shape. Using the specific binding of the anti-HER2 antibody to HER2, the anti-HER2 antibody was modified on MNS, and HER2 was effectively isolated and purified from the serum samples. Signal amplification was performed with ascorbate oxidase (AAO)-modified Co_3_O_4_ nanoparticles (Co_3_O_4_ NPs) and HER2 aptamers. The detection of HER2 is based on the reduction of the photocurrent intensity. The selectivity and specificity of the sensor were detected using human IgG, CEA, BACE1, p53 and human IgM, which confirmed its good selectivity and specificity. The linear range was 1 pg/mL~1 ng/mL, and the detection limit was 0.026 pg/mL [90].

## 5. Conclusions and Future Perspectives

The progresses and challenges in biosensor construction have facilitated the analysis of cancer biomarkers. There is no doubt that advances in biosensor technology will help in the establishment of bedside medical diagnostic devices. This review briefly introduced some commonly used sensor types for the detection of common tumor markers, such as electrochemical sensors, optical sensors, piezoelectric sensors, etc. The performance of these sensors is summarized in Table 3, including the operating linear range and detection limit of the sensors. Among them, the electrochemical sensors can detect common tumor markers, such as CEA, NSE, PSA, SCCA and CA19-9. Especially, in the detection of gastric cancer marker CEA, a variety of electrochemical sensor technologies have emerged combined with a variety of new materials, such as graphene oxide nanomaterials, chelated Eu^3+^ materials, etc., with a fast analysis speed, good selectivity and high sensitivity. It is helpful for early diagnosis, improving the treatment effect of patients and improving the quality of life. In addition, optical biosensors play an important role in the clinical diagnostics, drug discovery, etc. Generally speaking, optical biosensors have the advantages of high sensitivity, good stability and high reliability. Compared with ordinary electrochemical biosensors, they have higher accuracy and more important anti-interference ability. Additionally, in the experiment using a piezoelectric biosensor to detect PSA, since protamine can bind to fibrinogen, adding protamine to plasma can improve the detected LOD value.

However, it is still a great challenge to apply biosensors in the early clinical diagnosis and prognosis monitoring under practical demands. First, biomarkers are highly complex in organisms, and the detection of biomarkers cannot determine the presence of cancer, and environmental and genetic factors should also be considered. Second, due to the crossover of cancer cell genes, it is difficult to identify the type of cancer only by detecting the biomarkers, which is not helpful for early clinical diagnosis and early treatment. Finally, taking optical sensors as an example, most of the ones circulating on the market are still bulky and expensive. Therefore, the performance characteristics of biosensors need to be further improved, including reusability, stability and compatibility with biological fluids. Furthermore, they should be more integrated, miniaturized and portable in the future. If these issues are addressed, biosensors can be used to diagnose and manage cancer at a low cost in clinical laboratories and hospitals.

**Table 3:** A summary of the development of biosensors for common biomarkers.

Target	Method Type	Linear Range	Detection Limit	Reference
NSE	Electrochemical biosensor	10–500 ng·mL^−1^	0.133 ng·mL^−1^	[38]
NSE	Electrochemical biosensor	0.1–0.2 ng·mL^−1^	0.05 ng·mL^−1^	[37]
NSE	Optical biosensor	0.1–1000 ng·mL^−1^	0.05 ng·mL^−1^	[31]
NSE	Optical biosensor	-	1.0 pg·mL^−1^	[32]
NSE	photoelectrochemical biosensors	0.1 ng·mL^−1^–1000 ng·mL^−1^	0.05 ng·mL^−1^	[31]
NSE	photoelectrochemical biosensors	0.1 pg·mL^−1^–50 ng·mL^−1^	0.07 pg·mL^−1^	[33]
NSE	aptasensor	1–100 ng·mL^−1^	0.1 ng·mL^−1^	[36]
CEA	Electrochemical biosensor	0.001–100 ng·mL^−1^	0.27 pg·mL^−1^	[39]
CEA	Optical biosensor	10^−1^–10^3^ ng·mL^−1^	0.1 ng·mL^−1^	[40]
CEA	Optical biosensor	-	<100 fM	[42]
CEA	SERS	0.0001–100.0 ng·mL^−1^	0.033 pg·mL^−1^	[91]
CEA	Fluorescence	0.1 to 100 ng·mL^−1^	0.055 ng·mL^−1^	[44]
CEA	Photoelectrochemical sensors		0.14 pg·mL^−1^	[46]
CEA	Piezoelectric biosensor	1.5μg·ml^−1^–30 ug·ml^−1^	1.5 μg·ml^−1^	[48]
CEA	Aptasensors	0.25–1.5 nM	0.5 nM	[49]
CEA	Aptasensors	2–45 ng·mL^−1^	0.24 ng·mL^−1^	[50]
PSA	Electrochemical biosensor	5 pg·mL^−1^–20 ng·mL^−1^	0.33 pg·mL^−1^	[52]
PSA	Optical biosensor	1–50 ng·mL^−1^	0.5 ng·mL^−1^	[54]
PSA	Optical biosensor	50 fg·mL^−1^–10 ng·mL^−1^	21 fg·mL^−1^	[53]
PSA	Optical biosensor	1.0–250 ng·mL^−1^	0.72 ng·mL^−1^	[55]
PSA	Fluorescence	1 pg·mL^−1^–20 ng·mL^−1^	0.3 pg·mL^−1^	[56]
PSA	Fluorescence	1 × 10^−12^–1 × 10^−9^ g·mL^−1^	3 × 10^−13^ g·mL^−1^	[57]
PSA	Photoelectrochemical sensors	0.001 to 50 ng·mL^−1^	0.25 pg·mL^−1^	[58]
PSA	Photoelectrochemical sensors	0.005 to 20 ng·mL^−1^	0.0015 ng·mL^−1^	[59]
PSA	QCM	-	48 pg·mL^−1^	[60]
PSA	QCM	-	112 pg·mL^−1^	[61]
PSA	Aptasensors	0.005–100 ng·mL^−1^	1.75 pg·mL^−1^, 0.39 pg·mL^−1^	[62]
PSA	Aptasensors	0.01 to 50 ng·mL^−1^	0.01 ng·mL^−1^	[63]
SCCA	Electrochemical biosensor	0.0001–1 ng·mL^−1^ 1–30 ng·mL^−1^	25 fg·mL^−1^	[64]
SCCA	Electrochemical biosensor	5.0 p·mL^−1^–15.0 ng·mL^−1^	2.34 pg·mL^−1^	[65]
SCCA	Optical biosensor	0.001–75 ng·mL^−1^	0.22 pg·mL^−1^	[67]
SCCA	Photoelectrochemical sensors	0.001–75 ng·mL^−1^	0.3 pg·mL^−1^	[68]
CA19-9	Electrochemical biosensor	6.5–520 U·mL^−1^	0.26 U·mL^−1^	[69]
CA19-9	Electrochemical biosensor	0.0001–10 U·mL^−1^	31 μU·mL^−1^	[70]
CA19-9	SERS	-	1.3 × 10^−3^ U·mL^−1^	[71]
CA19-9	Photoelectrochemical sensors	0.1–1000 U·mL^−1^	0.01 U·mL^−1^	[72]
CA19-9	Piezoelectric biosensor	12.5–270.0 U·mL^−1^	-	[73]
TP53	Electrochemical biosensor	0.01–2 pg·mL^−1^	3 fg·mL^−1^	[75]
TP53	Electrochemical biosensor	20–1000 fg·mL^−1^	4 fg·mL^−1^	[76]
TP53	Fluorescence	50 pM–2 nM	8 pM	[78]
TP53	Photoelectrochemical biosensors	25 aM–2.5 pM	-	[80]
TP53	Piezoelectric biosensor	-	0.12 M	[81]
ATP6 AP1	QCM	-	1.73 × 10^−2^ mg	[84]
CA15-3	Electrochemical biosensor(cyclic voltammetry)	2.0~240 U·mL^−1^	0.64 U·mL^−1^	[86]
CA15-3	Electrochemical biosensor(PEDOT)	0.01~1000 U·mL^−1^	3.34 U·mL^−1^	[86]
HER2	photoelectrochemical sensor	1 pg·mL^−1^–1 ng·mL^−1^	0.026 pg·mL^−1^	[90]
AMF	electrochemical biosensor		43 fM	[92]
CYFRA-21	electrochemical biosensor		10^−2^ pM	[92]

## Figures and Tables

**Table 1:** Cancers and the relevant tumor markers. Reprinted with permission from [2].

Cancer	Relevant Tumor Markers
Neuroendocrine tumors	NSE
Gastrointestinal tumors	CEA, CA19-9
Prostate cancer	PSA, TP53
Cervical cancer	SCCA
Breast cancer	CA19-9, TP53
Epithelial ovarian tumors	CEA
Liver cancer	CEA, SCCA
Colorectal and pancreatic cancer	CA19-9
Lung cancer	CEA, CA19-9, NSE, SCCA

**Table 2:** Several tumor markers and their normal levels.

Tumor Markers	Thresholds
NSE	12.5 mg/L
PSA	4 ug/L
SCCA	1.5 ug/L
CEA	3 ug/L
CA19-9	37 U/L

## Data Availability

Not available.
